# Anisotropic scrunching of SMC with a baton-pass mechanism

**DOI:** 10.1038/s42003-024-06557-z

**Published:** 2024-07-19

**Authors:** Kyoung-Wook Moon, Do-Gyun Kim, Je-Kyung Ryu

**Affiliations:** 1 Department of Physics and Astronomy, Seoul National University, Seoul, South Korea; 2 Institute of Applied Physics of Seoul National University, Seoul, South Korea; 3 Institute of Molecular Biology and Genetics, Seoul National University, Seoul, South Korea; 4 Department of Biological Sciences, Seoul National University, Seoul, South Korea; 5 Interdisciplinary Program in Neuroscience, Seoul National University, Seoul, South Korea

**Keywords:** Atomic force microscopy, Single-molecule biophysics

## Abstract

DNA-loop extrusion is considered to be a universal principle of structural maintenance of chromosome (SMC) proteins with regard to chromosome organization. Despite recent advancements in structural dynamics studies that involve the use of cryogenic-electron microscopy (Cryo-EM), atomic force microscopy (AFM), etc., the precise molecular mechanism underlying DNA-loop extrusion by SMC proteins remains the subject of ongoing discussions. In this context, we propose a scrunching model that incorporates the anisotropic motion of SMC folding with a baton-pass mechanism, offering a potential explanation of how a “DNA baton” is transferred from the hinge domain to a DNA pocket via an anisotropic hinge motion. This proposed model provides insights into how SMC proteins unidirectionally extrude DNA loops in the direction of loop elongation while also maintaining the stability of a DNA loop throughout the dynamic process of DNA-loop extrusion.

## Abstract (web summary)

This Perspective discusses DNA-loop extrusion generated by SMC. The model details how the “DNA baton” is transferred from the hinge domain to a DNA pocket through anisotropic hinge motion, ensuring loop stability and providing insights into the dynamic process of DNA-loop extrusion.

## Introduction

**Fig. 1:**
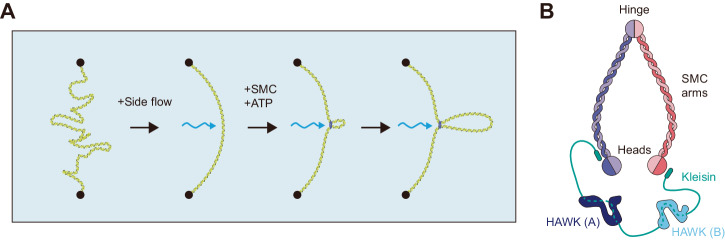
Drawing of the single-molecule DNA-loop extrusion experiment and a conserved SMC complex architecture. **A** Side flow experiments to show the loop extrusion process. **B** In eukaryotic cohesin and condensin complexes, three types of non-SMC subunits are found: kleisin subunits, HEAT repeat proteins associated with kleisins—HAWK (A) and HAWK (B). In yeast condensin, the kleisin subunit is Brn1, HAWK (A) is Ycs4, and HAWK (B) is Ycg1.

Structural maintenance of chromosome (SMC) complexes, including condensin, cohesin, and the SMC5/6 complex, play a crucial role in genome organization^1–5^. These complexes are present in various species and are believed to extrude DNA loops through ATP hydrolysis, serving as a fundamental genome organization mechanism^6,7^. Physiological evidence supporting this mechanism has been provided by sequencing-based technologies such as the high-throughput chromosome conformation capture technique (Hi-C), which measures the frequency of DNA contacts in genomes^8–14^. In vitro single-molecule experiments have successfully reconstituted the process of DNA-loop extrusion using different eukaryotic SMCs, such as *Saccharomyces cerevisiae* condensin^15,16^, *Homo sapiens* cohesin^17,18^, *Saccharomyces pombe* cohesin^19^, *Xenopus laevis* cohesin and condensin^20^, and the *S. cerevisiae* SMC5/6 complex^21^. These experiments have shown a common DNA-loop extrusion feature: the gradual growth of DNA loops in an ATP hydrolysis-dependent manner, accompanied by a perpendicular flow relative to the DNA, suggesting a conserved principle of various SMCs (Fig. 1A).

The SMC protein family exhibits a highly conserved architecture across organisms, from bacteria to humans (Fig. 1B). One distinguishing feature of the SMC protein family is the formation of an SMC-kleisin ring structure, which connects the SMC heads with a kleisin subunit. Additional two Huntingtin, EF3, PP2A, TOR1 (HEAT) repeat domains are attached to the kleisin subunit in eukaryotic cohesin and condensin. In this paper, we refer to yeast condensin subunits as follows: Brn1 as the kleisin, Ycs4 as HAWK (A), and Ycg1 as HAWK (B). The protein complexes and corresponding structures have been extensively studied; however, the molecular mechanism by which SMC extrudes a DNA loop remains a topic of much research^22–27^.

A scrunching model, in which the SMC-arm transforms from the extended configuration to a more compacted configuration for DNA-loop extrusion, is one of the most convincing working models^16,28–30^ (various models are described in Supplementary Table 1). However, how SMC unidirectionally extrudes the DNA loop in the direction of loop elongation and how the DNA loop can be consistently maintained during the dynamic extrusion cycle are challenges to be overcome to understand the model. To address these issues here, we review the current scrunching model by explaining how it is supported by experiments and simulations, after which we suggest a revised anisotropic scrunching model with a baton-pass mechanism. This review is based on studies of yeast condensin, as yeast condensin has been studied extensively using a variety of techniques, such as single-molecule fluorescence imaging, cryogenic-electron microscopy (cryo-EM), atomic force microscopy (AFM), magnetic tweezers (MT), cross-linking experiments, and molecular dynamic simulations, among others.

## Anisotropic extension-folding of SMC arms drives DNA-loop extrusion steps

**Fig. 2:**
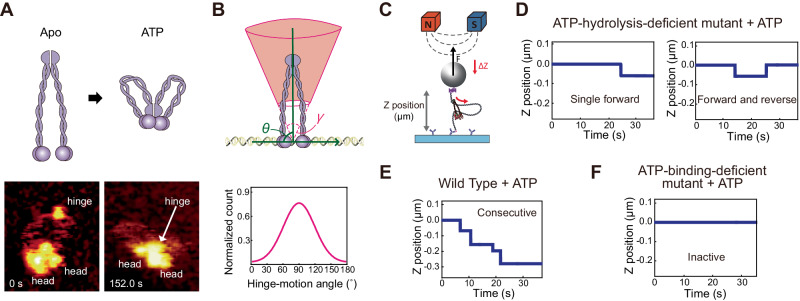
Various models of the conformational changes of SMC arms. **A** Illustration of the hinge-released state and the hinge-engaged state supported by HS AFM. Permissioned from ref. ^16^. **B** A truncated reachable cone region with a solid angle after the anisotropic hinge motion and the distribution of the hinge-motion angle with respect to the tangential line of DNA. **C** Schematic of magnetic tweezers experiments to monitor stepwise DNA-loop extrusion. The DNA length is defined by the end-to-end distance of the magnetic beads from the surface to the ends of the DNA to visualize the DNA end-to-end length. When condensin generates steps during the loop extrusion process, the bead position is lowered to a point that matches the step size of the loop. **D** Graphs of the appearance of a single forward step (left) or single forward and reverse steps (right) in an ATP hydrolysis-deficient mutant, an EQ mutant with ATP. **E** Graph of the occurrence of consecutive steps in wild-type condensin with ATP hydrolysis. **F** Graph of the inactive state in an ATP-binding-deficient mutant, a Q-loop mutant without ATP. Permission from ref. ^30^. **D**–**F** Based on the experimental data, we drew the traces to highlight stepwise DNA-loop extrusion traces.

In a recent experiment using high-speed AFM (HS AFM), researchers uncovered the conformational changes exhibited by yeast condensin, transitioning between the hinge-released state (where the hinge is significantly distant from the heads via the extension of SMC arms) and the hinge-engaged state (where the hinge is in close proximity to the heads via the folding of SMC arms) (Fig. 2A)^16^. Surprisingly, previous AFM experiments showed the anisotropic motion of the hinge through the extension-folding of SMC arms, meaning that the hinge preferentially moves in a particular direction instead of by isotropic motion^31^. The AFM data showed that the hinge motion was orthogonal to the tangent of the DNA, displaying an angle variation with a mean of 90° and a standard deviation of 30°. This angle variation adhered to a normal distribution, indicative of a constrained hinge motion, as opposed to a uniform distribution which would suggest equal movement in all directions (isotropic motion) (Fig. 2B)^29^. The hinge motion is restricted because the two SMC arms of the rigid upper and lower coiled-coil structures are coupled. From the distribution of the hinge motion, one can define a solid angle using the width of the hinge-motion angle distributions (~$60^\circ$) and the height from the hinge-engaged state (~10 nm) to the hinge-released state (~40 nm), which describes the hinge-reachable upside-down truncated cone and the anisotropic extension-folding of the SMC arms. Earlier work found that this anisotropic motion is very closely related to the elongational direction of the DNA-loop extrusion process (see “Origin of the directional extrusion activity”).

The molecular mechanism by which the ATP hydrolysis cycle is coupled with the extrusion step was also revealed by AFM and magnetic tweezers using a mutant lacking ATP hydrolysis capability (EQ mutant), which is unable to hydrolyze ATP but allows ATP stably to bind to it^16,30^ (Fig. 2A, C, D). A transition from the hinge-released state to the hinge-engaged state was detected by HS AFM, consistent with recent studies of the Cryo-EM structures of MukBEF and human/yeast cohesin, where the ATP-bound hinge-engaged state was also observed^32–35^. Moreover, the magnetic tweezers experiment showed that the EQ mutant with ATP generates a single forward step, or a forward step followed by a reverse step (Fig. 2D). The concept of “forward” and “reverse” steps refers to the process whereby the end-to-end distance of DNA is decreased (“forward step”) or increased (“reverse step”). In contrast, wild-type condensin, which can cyclically hydrolyze ATP, exhibited multiple consecutive steps (Fig. 2E), and the mutant lacking ATP-binding capability did not show any steps (Fig. 2F). These results indicate that the process of step generation is induced by SMC folding from the hinge-released state to the hinge-engaged state via ATP binding, with ATP hydrolysis a necessary step in the subsequent rounds of DNA-loop extrusion. Although how the hinge-head decoupling is coupled to ATP hydrolysis requires further experimentation, at this point the most relevant evidence of this process is the previous HS AFM result showing the hinge-released state in the absence of ATP^16^.

## Evidence of the scrunching model

Initially, the scrunching model was proposed to depict the process of initial bacterial transcription, wherein the RNA polymerase remains stationary on the promoter DNA and draws the downstream DNA towards itself and past its active center^36,37^. Recent yeast condensin and human cohesin studies suggested the scrunching model as a DNA-loop extrusion mechanism^16,28,29,38^. In this model, the hinge is a motoring site while Ycg1 is an anchoring domain that constantly anchors onto DNA. Interestingly, the scale of large conformational changes measured by the hinge motion via the folding of the long SMC arms is similar to the step sizes recently measured by magnetic tweezers^30^. Moreover, molecular dynamic simulations supported the scrunching model by assuming conformational changes from the hinge-released to hinge-engaged shapes (shown as 26 nm from the head to the hinge)^28–30^. These simulated results match the loop extrusion velocity and the observed step sizes from 17 nm (60 bp) to 40 nm (~220 bp) depending on the low applied tension (1.0–0.2 pN). The measured or simulated force-dependent DNA-loop extrusion step sizes show DNA fluctuation dependency.

Condensin has a low stalling force (1–2 pN) due to the generation of a large step size with a fixed amount of ATP hydrolysis energy (~50 pN·nm). At low force levels, condensin extrudes DNA loops in steps up to hundreds of base pairs, which exceeds the measured conformational changes, while at higher force levels, the step sizes becomes as large as the conformational changes of SMC, indicating that condensin is a unique motor protein that uses large conformational changes and reels along a flexible polymer (DNA with a persistence length of ${L}_{P}=50\,{{{{\rm{nm}}}}}$), while other motor proteins such as dynein, kinesin, and myosin walk along stiff filaments (actin filaments and microtubules, ${L}_{P} > 1\,\upmu {{{{\rm{m}}}}}$)^39^. The low loop extrusion stalling force (~1 pN)^30,40^ aligns well with the results of clamping experiments using optical tweezers, demonstrating that the hinge-head motion cannot overcome forces above 1 pN, while the head-head motion can overcome forces up to 15 pN^41^. Furthermore, another MD simulation study of the anisotropic hinge movement in the scrunching model allowed an explanation of the unidirectional loop elongation process, validating the scrunching model^29^. However, a MD simulation based on the DNA-segment capture model showed similar low stalling force and similar large step sizes using relatively small conformational changes^42^. Therefore, future work is required to understand precisely how conformational changes are involved in the DNA-loop extrusion process.

## Three distinct DNA-binding sites

**Fig. 3:**
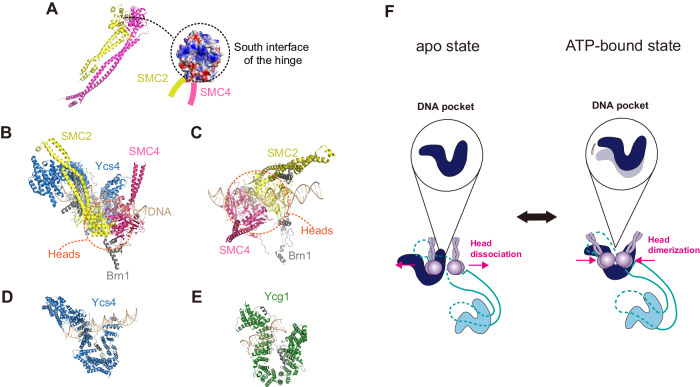
Atomic structures of yeast condensin and a drawing of the reconstructed movements of non-SMC subunits. **A** Structure of the hinge and electrostatic surface charge distribution of the hinge (PDB: 6WG3, 7QEN, 4RSI). **B** Globular domain of yeast condensin in the ATP-bound state (dimerized SMC heads depicted by the dotted line). **C** Dimerized SMC heads that bind to DNA. **D** Ycs4 bound to DNA functions as a DNA pocket to stabilize DNA-loop extrusion. **E** Ycg1 bound to DNA that anchors DNA with Brn1 (gray) using a safety belt mechanism. **F** Movement of the DNA pocket and the dimerization of the heads via ATP binding.

Although the current scrunching model requires the existence of at least two DNA-binding sites (the hinge for motoring action and Ycg1/Brn1 for anchoring), we propose that one additional DNA-binding pocket is essential to maintain a stable DNA-loop structure during the dynamic DNA-loop extrusion process. Based on current cryo-EM studies and cross-linking experiments, the heads and Ycs4 appear to coordinate dynamically to provide a third DNA-binding pocket to hold a DNA loop as the hinge targets a new DNA-binding site^32,33,35,43,44^. The inner region of the hinge observed in structural studies has a positively charged surface that can bind to DNA (Fig. 3A). In addition, recent mutagenesis studies of the hinge region have shown impaired DNA-loop extrusion activity, supporting the hypothesis that DNA-hinge binding is involved in the DNA-loop extrusion process^38^. This indicates that the south interface of the hinge region is likely used to interact with DNA^39^. The SMC heads within the complexes are asymmetrically dimerized via ATP binding, and this dimerization process creates a site for DNA binding in conjunction with Ycs4 (Fig. 3B, C)^43,45^. In particular, the groove region of Ycs4 on the left and the inner concave region of Ycg1 of yeast condensin also serve as DNA-binding sites (Fig. 3D, E)^43^. The formation of this third DNA-binding site is likely facilitated by the coordinated action of Ycs4 and Brn1, and the ATP hydrolysis cycle in the SMC heads domains is thought to contribute to the organization of the DNA pocket in this context (Fig. 3F)^35,43^. We suggest that this coordination is essential for maintaining the stability of the DNA loop and preventing disruptions, particularly when the motor domain of the complex targets a new DNA region. Without this third site, the DNA region bound to the motor domain could easily slip. In recent structural investigations, significant DNA-interaction sites within the SMC complex have been identified, exhibiting a high degree of conservation across various SMCs^22,23,27^.

## Anisotropic scrunching model with a baton-pass mechanism

**Fig. 4:**
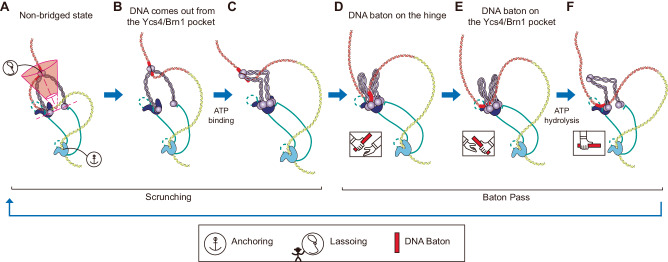
Schematic of the extended anisotropic scrunching model with a baton-pass mechanism. **A** Ycg1/Brn1 anchors DNA and the hinge captures the DNA baton, which is in a truncated cone area within the anisotropic region. **B** The DNA segment held by the Ycs4/Brn1 pocket can escape due to the space between the pocket and the neck of SMC2. **C** When ATP binds to the SMC heads, the heads are dimerized and the arms of the SMC are folded. **D** The Ycs4/Brn1 DNA pocket is closed, and **E** the hinge passes the DNA baton to the DNA pocket. **F** After ATP hydrolysis, the DNA pocket is open to release the DNA baton and the SMC arms are extended.

Here, we suggest an anisotropic scrunching model with a baton-pass mechanism that relies on the coordinated movements of the hinge and non-SMC subunits (Fig. 4). Initially, the condensin complex is anchored at a specific DNA region by Ycg1/Brn1, establishing a stable starting point^15,35,38,46^. The hinge region then targets and binds to a DNA region akin a lasso by moving anisotropically in an orthogonal direction with respect to the tangent DNA line where the heads are located on the DNA^16^ (Fig. 4A)^29^. The DNA segment on the Ycs4/Brn1 can be released from the Ycs4/Brn1 pocket because it is not closed in an apo state (Fig. 4B). In the baton-pass mechanism, the targeted DNA region acts as a “DNA baton” that must be passed to the non-SMC subunits, and this process appears to be regulated by ATP binding and hydrolysis. ATP binding induces head dimerization, creating a dynamic DNA-binding site on the aligned dimerized heads with Ycs4 (Figs. 3B and 4C). The hinge is then favorably engaged with the aligned dimerized heads/Ycs4/Brn1, inducing a transfer of the DNA baton from the hinge region to the dimerized heads/Ycs4 in the aligned state (Fig. 4D, E) via SMC folding due to the higher affinity of the aligned state. Lastly, ATP hydrolysis occurs, and the consecutive rounds proceed, extruding the loop continuously (Fig. 4F).

For the baton-pass mechanism, the binding and hydrolysis of ATP are synchronized with the transfer and capture of DNA from the hinge to the third DNA pocket through the structural dynamics of the heads/Ycs4/Brn1. ATP binding induces the dimerization of the heads and the folding of SMC arms. The DNA baton captured on the hinge is transferred to the heads/Ycs4/Brn1. This process is triggered by the higher affinity of the dimerized heads compared to the hinge. DNA binding can then induce the alignment of the Ycs4/Brn1 configuration with the heads along with the DNA baton locates in between the neck of SMC2 and Ycs4. ATP hydrolysis induces the dissociation of the heads, and the DNA baton becomes entrapped by the Ycs4/Brn1 pocket, with the Smc2 neck following the DNA-binding site in Ycs4/Brn1, causing the loop extrusion process to be irreversible because a non-topological loop is formed via the Ycg1/Brn1 anchoring site and the Ycs4/Brn1 pocket. This can explain the consecutive steps observed via multiple ATP hydrolysis in magnetic tweezers experiments as the effect of ATP binding causes reversible step generation^28^.

After the hinge release step, the hinge targets a new DNA baton. Although the Ycs4/Brn1 pocket has relatively weak DNA-binding affinity compared to the dimerized heads when aligned with Ycs4/Brn1, this interaction is sufficient to maintain the non-topological loop structure. In addition, due to this weak interaction, we speculate that once the hinge targets a new DNA baton, the previously entrapped DNA baton in the Ycs4/Brn1 pocket would be released through the small gap between Ycs4 and the Smc2 neck in the non-bridged state. The driving force behind this release could be the higher tension applied to the DNA string to in turn induce destabilization of the DNA baton interaction with the Ycs4/Brn1 pocket. Afterward, the next cycle continues when the new DNA baton is transferred from the hinge to the dimerized heads. Given that the binding affinity of the Ycs4/Brn1 binding pocket is modulated by head dimerization and dissociation, this modulation is utilized to synchronize the DNA interaction with the hinge and the interaction with Ycs4/Brn1 for the DNA baton-pass mechanism. To transfer the DNA baton from the hinge to the head, dimerized heads provide a DNA-interaction site with a higher affinity than the hinge, whereas dissociated heads decrease the DNA-binding affinity with the Ycs4/Brn1 pocket. With synchronization, DNA would not preferentially bind to the Ycs4/Brn1 pocket over the hinge initially.

## Discussion

### Origin of the directional extrusion activity

How can our model explain the elongational directionality of DNA-loop extrusion? Although the original scrunching model cannot aptly explain the mechanism of directional motion, the anisotropic scrunching model can explain this mechanism^44^. A recent study using AFM and a MD simulation showed that the anisotropic hinge motion can explain this mechanism^31^. From the recent AFM study, based on the distributions of the hinge motion observed in AFM images, a hinge-reachable region could be defined as a truncated cone shape with a solid angle using the width of the distribution. The MD simulation showed double the number of forward steps compared to reverse steps by assuming that the anisotropic hinge motion is constrained into the truncated cone shape (Fig. 2B). This study modeled the forward step as generated when the hinge captures the outside of the DNA loop to elongate, with the reverse step generated when the hinge captures the inside of the DNA loop and transfers it to induce shortening of the loop size. However, a different hinge-searching angle showed a different ratio of the number of forward-to-reverse steps. In an extreme case, assuming an isotropic hinge-reachable region (i.e., a 4π steradian solid angle), the extruded DNA-loop length showed random walk behavior (i.e., the number of forward and reverse steps were identical). These simulation results suggest that the anisotropic hinge motion is the origin of the directional motoring activity.

In addition, the difference in the molecular mechanism for the one-sided or two-sided DNA-loop extrusion cases can be explained by the DNA exchange of the anchoring site and the Ycs4/Brn1 pocket. Yeast condensin showed one-sided DNA-loop extrusion behavior (asymmetric DNA-loop extrusion), while cohesin showed two-sided DNA-loop extrusion behavior (symmetric DNA-loop extrusion). The occurrence of this process is possible when a DNA baton is captured in the HAWK (A) pocket after ATP hydrolysis if HAWK (B) does not strongly anchor DNA. It appears that Ycg1 (HAWK (B)) of the yeast condensin complex has strong DNA-binding affinity such that it can serve as an anchor, while STAG1 (HAWK (B)) in humans (or Scc3 in yeast) appears to have weaker DNA-binding affinity. Perhaps the CTCF interaction with STAG1 can enhance the DNA-binding affinity, similar to the Ycg1 case^47^.

### Stabilization of the DNA loop during the dynamic extrusion process

Because the DNA-loop extrusion process requires dynamic interactions between DNA and proteins at distinct sites, the DNA loop should be maintained once it is extruded without slippage, as slippage can induce shortening the length of the elongated loop. The baton-pass mechanism can explain how a stable DNA loop is maintained during the dynamic extrusion process through the holding of a dynamic loop using the third DNA-binding site and the anchoring domain. If two DNA-binding sites of SMC do not stably hold two distinct DNA segments that define a loop, SMC can easily lose its elongated loop structure, as loop extrusion should occur with the motoring site capturing a new DNA segment while simultaneously extruding the previously captured DNA. Hence, this process requires rapid DNA replacement at the motoring site to prevent DNA slippage during loop extrusion, particularly considering that Brownian motion of the DNA or protein can destabilize the loop structure. Accordingly, precise synchronization between SMC and DNA motion is essential.

The baton-pass mechanism with three-DNA-binding sites offers increased stability so as to maintain the DNA-loop structure. While one site anchors the DNA, the remaining two binding sites can serve to both target a new DNA region and hold the previously looped region by synchronously organizing the interaction between these two DNA-binding sites. The hinge and the Ycs4/Brn1 pocket are modulated by the dynamic opening and closing of the hinge region and the dimerization of the heads. Recent HS AFM studies have shown the dynamic opening and closing angle of the hinge region, suggesting a form of transient interaction between the hinge and DNA that depends on how open the hinge region is^16,48^. The transient interaction of the hinge and DNA could be used for the DNA baton transfer to the next DNA-binding pocket. Once the hinge is released after the DNA transfer step, another DNA-binding site with an anchoring site should hold the DNA baton to maintain the loop structure. In addition, ATP-binding-induced head dimerization provides a stable DNA-binding site that readily facilitates the transfer of the DNA baton from the hinge. While the earlier iteration of the scrunching model lacks the capability to elucidate the process by which the dynamic loop is maintained during motor actions, the baton-pass mechanism can bolster the scrunching model by addressing this inherent limitation.

### Mechanisms of roadblock bypassing

Recent single-molecule experiments have demonstrated that a fused-tripartite SMC-kleisin ring of cohesin bypasses through roadblocks much larger than the ring size, suggesting that the non-topological mode of SMC extrudes a DNA loop^49^ (Supplementary Fig. 1). Because the physiological loop size of the mammalian genome exceeds 100 kbp, this mechanism can explain how SMCs can generate such elongated loops by passing through many barriers formed by DNA-interacting proteins such as histones (distributed with a 200-bp period), transcription factors, and RNA polymerase, among others. To explain this mechanism, we speculate that the elongated loop structure is maintained by the synchronization between the pseudo-topological loop that arises due to the attachment of the hinge and the Ycg1/Brn1 anchor, with the non-topological loop held by the Ycs4/Brn1 pocket and Ycg1/Brn1 anchor (Fig. 4). In addition, DNA may bind to the outside of Brn1 at both the DNA pocket site and the anchoring site. If the hinge functions as a motor and passes DNA to the DNA pocket while Brn1 is holding DNA externally, the roadblock experiments are explainable. Further experiments should be conducted to clarify how the baton-pass mechanism allows for the bypassing of large roadblocks with a fused-tripartite SMC-kleisin ring.

### Concluding remarks

Over the past decade, numerous studies have shed light on the functional roles of SMC proteins in relation to the organization of genomes. The DNA-loop extrusion mechanism has emerged as a widely and strongly supported universal principle for SMC proteins. In addition, recent structural studies have made significant breakthroughs with regard to understanding the molecular mechanisms involved, although some aspects still require further study. From recently acquired structural data and molecular dynamics (MD) simulations, the anisotropic scrunching model can clarify the process by which SMC extrudes a DNA loop in a unidirectional fashion. Furthermore, the incorporation of the baton-pass mechanism provides insights into the transfer of a DNA baton from the hinge domain to a DNA pocket, consequently guaranteeing the stability of the DNA loop throughout the dynamic DNA-loop extrusion process. Looking ahead, we anticipate that the next decade will bring further clarity to the molecular mechanisms underlying SMC-mediated DNA-loop extrusion through advancements in cryo-tomography, advanced single-molecule observations, and structural dynamics studies. For example, liquid-phase HS-AFM imaging of the structural dynamics of SMCs during DNA-loop extrusion are able to provide hints. Moreover, simulations using the baton-pass mechanism by applying the third DNA-binding pocket in Ycs4/Brn1 also need to be explored in the future.

### Reporting summary

Further information on research design is available in the Nature Portfolio Reporting Summary linked to this article.

### Supplementary information

Supplementary Information

Reporting Summary
