# Supplementary material for: Anisotropic scrunching of SMC with a baton-pass mechanism
Source: Commun Biol. 2024 Jul 19;7:881. doi: 10.1038/s42003-024-06557-z (PMC11271495; doi:10.1038/s42003-024-06557-z)
Supplement: Supplementary file 1 — Supplementary Information [file 42003_2024_6557_MOESM1_ESM.docx]

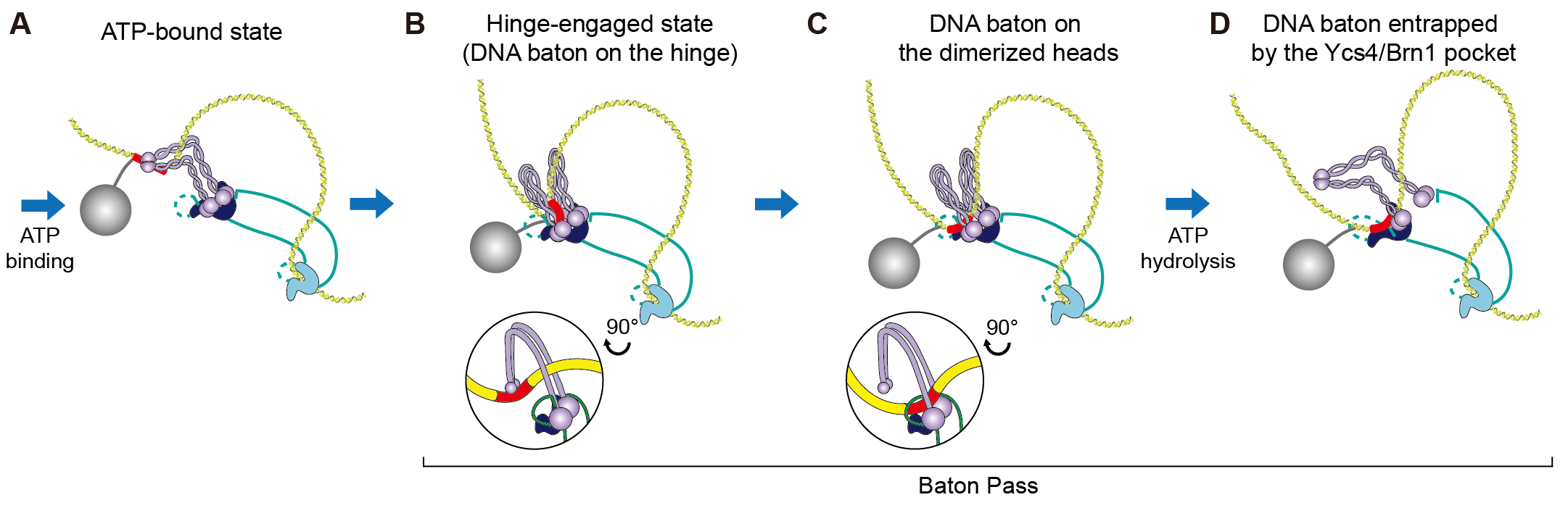


**Supplementary Figure 1. Schematic of the revised anisotropic scrunching model with a baton-pass mechanism to explain roadblock bypassing:** **(A)** When ATP binds to SMC heads, the heads are dimerized, the SMC arms start to fold, and the roadblock moves closer to the SMC heads. A loop is formed in a pseudo-topological manner. **(B)** Simultaneously, the Ycs4/Brn1 DNA pocket is closed, and **(C)** the hinge passes the DNA baton to the DNA pocket. At this moment, the DNA segment connected to the roadblock moves to the Ycs4/Brn1 pocket. **(D)** After ATP hydrolysis, the DNA pocket is open to release the DNA baton and the SMC arms start to extend. DNA-loop extrusion is able to occur via the roadblock.

| **Model** | | **Apo state** | **ATP binding state** | **After ATP hydrolysis** | **Step generation** | **Open questions** | **Reference** |
| --- | --- | --- | --- | --- | --- | --- | --- |
| DNA segment capture model | Conformation | Closed SMC arms | Heads dimerization SMC-arm opening | Heads disengagement SMC-arm zipping | SMC zipping transfers DNA | The driving force for the zipping of two SMC arms needs to be explored. | [1] |
|  | DNA-binding sites | Anchor site: Ycg1/Brn1 Motor site: Ycs4/Brn1 | SMC2 head/Ycs4/Brn1 | Hinge $\to$ Ycs4/Brn1 |  |  |  |
| Reel and Seal model | Conformation | Folded SMC arm | Heads dimerization Unfolded SMC arm Locking of Ycs4 & SMC2 neck | Heads disengagement Ycs4 (apart from the heads $\to$ closer to the heads with SMC arm folding) | Folding of SMC arms induces Ycs4 attached to the heads | The mechanism by which ATP-binding step generates a single-loop extrusion step needs to be explained. | [2] |
|  | DNA-binding sites | Anchor site: Ycg1/Brn1 Motor site: Ycs4/Brn1 | Ycs4/Brn1 Hinge | Hinge $\to$ Ycs4/Brn1 |  |  |  |
| Anisotropic scrunching model with a baton-pass mechanism | Conformation | Hinge-released | Heads dimerization Hinge-engaged Locking of Ycs4 & SMC2 neck | Heads disengagement Ycs4 opening (along with the heads) |  | Structural dynamics of the baton-pass mechanism needs to be explored. |  |
|  | DNA-binding sites | Anchor site: Ycg1/Brn1 Motor site: Hinge | Hinge $\to$ Ycs4/Brn1 Ycs4/Brn1 | Baton-pass: Ycs4/Brn1 |  |  |  |

**Supplementary Table 1. Comparative study of DNA-loop extrusion models in the apo, ATP binding, and post-hydrolysis states**

**Reference**

1. Marko, J.F. *et al.* (2019) DNA-segment-capture model for loop extrusion by structural maintenance of chromosome (SMC) protein complexes. *Nucleic Acids Res* 47, 6956–6972

2. Dekker, C. *et al.* (2023) How do molecular motors fold the genome? *Science (2023)* 382, 646–648
